# Molecular targets of Yangyin Fuzheng Jiedu Prescription in the treatment of hepatocellular carcinoma based on network pharmacology analysis

**DOI:** 10.1186/s12935-020-01596-y

**Published:** 2020-11-09

**Authors:** Fengna Yan, Miaomiao Feng, Xinhui Wang, Peng Wang, Yuqing Xie, Xiaoli Liu, Weihong Li, Zhiyun Yang

**Affiliations:** 1 Center for Integrative Medicine, Beijing Ditan Hospital Capital Medical University, No. 8, Jingshun East Street, Chaoyang District, Beijing, 100015 People’s Republic of China; 2 Spleen Stomach Institute, Longhua Hospital Shanghai University of Traditional Chinese Medicine, Shanghai, 200030 People’s Republic of China; 3 School of Nursing, Beijing University of Chinese Medicine, No. 11, Bei San Huan East Road, Chaoyang District, Beijing, 100029 People’s Republic of China

**Keywords:** Hepatocellular carcinoma, Yangyin fuzheng jiedu prescription, Cumulative survival, Network pharmacology, Apoptosis, Migration, Invasion, Proliferation

## Abstract

**Background:**

Yangyin Fuzheng Jiedu Prescription (YFJP) is a traditional Chinese medicine (TCM) indicated for the treatment of hepatocellular carcinoma (HCC). Its potential targets and molecular mechanisms are not clear. Therefore, this study intends to explore the molecular mechanism of YFJP based on network pharmacology analysis and in vitro validation.

**Methods and results:**

Through univariate and multivariate analyses and survival analysis in HCC patients with or without YFJP treatment we found that drinking alcohol, alfafeto protein ≥ 400 ng/l, baseline portal vein tumor thrombus and total bilirubin level ≥ 18.8 μM) were independent risk factors for poor prognosis, while red blood cell count ≥ 4 × 10^9^/l and TCM treatment were independent protective factors. Besides, YFJP prolonged the cumulative survival of HCC patients. Using online pharmacological methods, we obtained 58 relevant compounds and molecular 53 targets. By using scratch test, Transwell assay, EdU assay, and TUNEL staining, we found that YFJP-containing serum repressed the migration, invasion and proliferation of HCC cells in vitro, and induced cell apoptosis. Moreover, YFJP diminished the gene expression of TP53, CCND1, p-EGFR, EGF, VEGFA, JUN, IL6, COX-2, AKT1, and MAPK1 in HCC cells, but elevated the expression of ESR1 and CASP3.

**Conclusions:**

Taken together, results showed that YFJP attenuated HCC progression through mediating effects on HCC-related genes.

## Background

Human liver cancers, including hepatocellular carcinoma (HCC) and intra-hepatic cholangiocarcinoma, are commonly diagnosed at advanced stages, resulting in unfavorable prognosis [1]. Among primary liver cancers, nearly 90% of cases are HCC, which frequently occurs in the setting of chronic inflammation [2]. HCC has thus become one of the most frequently occurring malignancies throughout the globe, accompanied by a high mortality and an increasing incidence rate in many countries [3, 4]. It has been reported that hepatitis B virus infection is a leading risk factor for developing HCC, particularly in East Asia [5]. Present surgical options are fairly effective low grade stages for HCC, but the 5-year overall survival rate for high grade stages is only 50–70% [6]. Recent evidence has strongly supported the application of traditional Chinese medicine (TCM) to combat HCC in reducing poor prognosis and recurrence through suppressing cancer cell invasion and metastasis [7].

Increasing numbers of studies have documented the amelioration of TCM of detrimental events accompanying surgery, chemotherapy or radiotherapy anti-cancer treatments, which is partly ascribed to enhanced activation of the immune-system and consequent facilitation of cancer cell apoptosis [8, 9]. It is interesting to note that Yangyin Fuzheng Decoction Prescription (YFDP) is a traditional Chinese compound medicine processed from native Chinese herbs, with anti-tumor properties that are the subject of active investigation [10]. In addition, the prescription of Fuzheng Jiedusan has been investigated in the treatment of advanced gastric cancer, where it proved to augment therapeutic response, disease control, and life quality of patients [11]. This present study aims to identify the potential targets and molecular mechanisms associated with the antineoplastic properties of YFJP, which has been used clinically for many years in the treatment of liver cancers of the Center of Integrative Medicine, Beijing Ditan Hospital (Affiliated to Capital Medical University). The study based on this prescription has been registered in the clinical trial system (https://www.clinicaltrials.gov/) a registration number NCT02927626. Chemical constituents of YFJP have been investigated by high performance liquid chromatography (HPLC) in conjunction with mass spectrometry, but its therapeutic mechanism is still unclear. Of note, tumor protein p53 (TP53) and interleukin 6 (IL-6) have been highlighted as targets of TCM liver-regulating herbs, which exert effects through modulating various biological processes and inflammatory pathways [12]. Prior evidence has suggested roles for epidermal growth factor receptor (EGFR), estrogen receptor 1 (ESR1), and prostaglandin-endoperoxide synthase 2 (PTGS2) in the molecular mechanisms of *Astragalus membranaceus* (Huáng Qí, a flowering plant in the family Fabaceae used in TCM) and applied animal models to investigate its mechanism of action [13]. Moxibustion, another TCM method, has been reported to exercise its beneficial effects through modulating the production of epidermal growth factor (EGF) and the activity of AKT serine/threonine kinase 1 (AKT1) [14]. Moreover, biochemical investigated have identified mitogen-activated protein kinase 1 (MAPK1) and jun proto-oncogene (JUN) as the key targets underlying the efficacy of the Chinese patent medicine Zhixiong Capsule [15]. Similarly, the pro-angiogenic effects and mechanism of action of Shexiang Baoxin Pill (another commonly used TCM) have been attributed to production of pro-angiogenic factor vascular endothelial growth factor A (VEGFA) [16]. A bioinformatics investigation identified the caspase 3 (CASP3) as a pivotal target for the pharmacological mechanisms of Yinchenhao Decoction on hepatitis C, partly due to its potential to modulate pertinent biological pathways [17]. Notably, a prior study on the efficacy of the O-methylated flavone wogonin on HCC, indicated accelerated phosphorylation of cyclin D1 (CCND1) to be a factor in the suppression of tumor growth [18]. Therefore, the current study aimed to investigate the anti-tumor activity and the underlying mechanisms of YFJP associated with the bioinformatically identified potential target genes.

## Materials and methods

### Analysis of survival rate and influencing factors

#### Ethics statement

The current study was approved by the Committee of Ethics at Beijing Ditan Hospital, Capital Medical University. All patients singed informed consent on the use of their data in the study. This study did not pose risk to patients and did not harm patients’ right or health. All animal experiments were approved by local authorities and were performed following the Guide for the Care and Use of Laboratory Animals of the National Institutes of Health, USA.

#### Study subjects

From January 2008 to June 2017, a total of 477 HCC patients were enrolled in this study. We used 1:2 frequency matching by sex and age to compare the TCM users (n = 159) and non-TCM users (n = 318). The main inclusion criteria were as follows: The patients were confirmed by the western medical diagnostic criteria for primary liver cancer (the "*Specifications for the Diagnosis and Treatment of Primary Liver Cancer*" formally revised by the Ministry of Health of the People's Republic of China in 2017); The patients were followed up for more than one year; The patients were 18–80 years old, and of male and female gender. Exclusion criteria were as follows: Patients with viral infections, such as human immunodeficiency virus (HIV), metastatic liver cancer or other types of tumors, pregnant women, less than one year of follow-up, and incomplete record of clinical data and relevant examination results. Patients in the YFJP group were aged 27 to 77 years, with mean age of 57.216 ± 9.95 years, and patients in the non-YFJP group were aged 20 to 80 years, with mean age of 57.02 ± 11.03 years. All patients were routinely treated with antibacterial drugs, hemostasis, and enhanced liver protection treatment. Patients in the YFJP group were treated with YFJP the day after transarterial chemoembolization (TACE) treatment.

#### YFJP treatment regimen

*Glehnia littoralis F.Schmidt ex Miq.* (Chinese name: Beishashen, 15 g), *Ophiopogon japonicus (Thunb.) Ker Gawl.* (Chinese name: Maidong, 15 g), *Hedysarum multijugum Maxim.* (Chinese name: Huangqi, 20 g), *Atractylis macrocephala (Koidz.) Hand.-Mazz.* (Chinese name: Baizhu, 9 g), *Bupleurum chinensis DC.* (Chinese name: Chaihu, 9 g), *Sophora flavescens Aiton* (Chinese name: Kushen, 9 g), *Cynanchum paniculatum (Bunge) Kitagawa* (Chinese name: Xuchangqing, 12 g), and *Hedyotis diffusa Willd.* (Chinese name: Baihua Sheshecao, 20 g) were purchased from the Pharmacy of Traditional Chinese Medicine, Beijing Ditan Hospital. Patients in the YFJP group received comprehensive treatment with Western medicine supplemented with YFJP for ≥ 2 months; they took an oral dose of YFJP for two days after decocting in water; while the non-YFJP group received only comprehensive treatment as usual according to Western medicine.

#### Follow up

Patients were followed up after treatment, mainly by telephone interview or chart review. The follow-up lasted for at least one year, and the 5-year cumulative survival rate of patients was recorded. The survival time of patients was recorded, and survival curves were plotted by Kaplan–Meier analysis with and without YFJP as variables. Results were tested by univariate and multivariate statistical analyses.

### HPLC sample preparation

In the previous experiments, we studied the effects of different solvent dosage, solvent volume, extraction temperature, extraction time and times on the extraction amount of total polysaccharides, total flavonoids and total saponins in YFJP. The final preparation conditions of YFJP were determined as follows: a single dose of 10 times the volume of double distilled water as the extraction solvent, extracting at 75 ℃ for 45 min and 30 min, respectively. The retention rate of total flavonoids, total polysaccharides and total saponins of the extract were all above 80%, which indicated that the extraction process was feasible.

TCMs *Glehnia littoralis F.Schmidt ex Miq.* (15 g), *Ophiopogon japonicus (Thunb.) Ker Gawl.* (15 g), *Hedysarum multijugum Maxim.* (20 g), *Atractylis macrocephala (Koidz.) Hand.-Mazz.* (9 g), *Bupleurum chinensis DC.* (9 g), *Sophora flavescens Aiton* (9 g), *Cynanchum paniculatum (Bunge) Kitagawa* (12 g), and *Hedyotis diffusa Willd.* (20 g) were mixed in 100 ml double distilled water and successively decocted by boiling for 45 min and 30 min. Next, the mixture was filtered and concentrated by evaporation to 73 ml, corresponding to 1.5 g of YFJP per 1 ml of the solution. 0.2 ml concentrated YFJP decoction was added with 95% ethanol, filtered to remove floccular sediment, concentrated and dried, and then dissolved with 50 ml methanol. After centrifugation, the supernatant passed through a 0.22 μm microporous membrane, and the filtrate was used as the YFJP test solution.

The preparation of the reference solution was carried out as follows: a sample of mullein isoflavone glucoside reference substance was precisely weighed, and was dissolved in methanol to a concentration of 50 μg/ml. The phenolic reference substance paeonol was dissolved in methanol to a concentration of 20 μg/ml.

### Chromatographic conditions

The column measuring  4.5 cm × 5 mm internal diameter contained octadecyl silane bonded silica gel was equipped with a 5 cm protective precolumn, and was kept at 30 ℃; The column temperature was 30 ℃. The injection volume was 20 μL. The binary mobile phase consisted of (A) acetonitrile, and (B) 0.2% formic acid in water, which was delivered at a total flow rate of XX ml/min in a gradient as follows 0—20 min, 5—40% A; 95—60% B; 20—40 min, 40—70% A; 60—30% B, with online ultraviolet detection at a wavelength of 260 nm. The number of theoretical plates was not less than 3000 based on the peak of the isoflavone glucoside peak.

### Network pharmacology analysis

#### Analysis for the compound components of YFJP

The TCMSP database (https://tcmspw.com/tcmsp.php) was used to retrieve the components of *Glehnia littoralis F.Schmidt ex Miq., Hedysarum multijugum Maxim., Atractylis macrocephala (Koidz.) Hand.-Mazz., Bupleurum chinensis DC., Sophora flavescens Aiton, Cynanchum paniculatum (Bunge) Kitagawa,* and *Hedyotis diffusa Willd.* in YFJP, and the screening conditions were set at OB ≥ 30% and DL ≥ 0.18. The BATMAN database (https://bionet.ncpsb.org/batman-tcm/) was used to search for compound components in *Ophiopogon japonicus (Thunb.) Ker Gawl.* (Score cutoff ≥ 20, *p* value ≥ 0.05). A total of 120 compounds were obtained, including eight compounds in *Glehnia littoralis F.Schmidt ex Miq.*, 22 compounds in *Ophiopogon japonicus (Thunb.) Ker Gawl.*, 20 compounds in *Hedysarum multijugum Maxim.*, seven compounds in *Atractylis macrocephala (Koidz.) Hand.-Mazz.*, 17 compounds in *Bupleurum chinensis DC.*, 45 compounds in Sophorae Flavescentis, six compounds in *Cynanchum paniculatum (Bunge) Kitagawa*, and seven compounds in *Hedyotis diffusa Willd.*

### Target analysis for the YFJP compounds

Targets corresponding to the compounds in YFJP were searched through the TCMSP and BATMAN databases. In addition, the official gene symbol corresponding to the target protein was searched (the “species” was limited to “Homo sapiens”) through the UniProtKB database (http: //www.uniprot/). A total of 411 targets were obtained, including 146 compounds corresponding to *Glehnia littoralis F.Schmidt ex Miq.*, 244 compounds corresponding to *Ophiopogon japonicus (Thunb.) Ker Gawl.*, 142 compounds corresponding to *Hedysarum multijugum Maxim.*, 16 compounds corresponding to *Atractylis macrocephala (Koidz.) Hand.-Mazz.*, 154 compounds corresponding to *Bupleurum chinensis DC.*, and 162 compounds corresponding to *Sophora flavescens Aiton*, 11 compounds corresponding to *Cynanchum paniculatum (Bunge) Kitagawa*, and 152 compounds corresponding to *Hedyotis diffusa Willd.*

#### Analysis for HCC-related targets

HCC-related targets were searched through the CTD (/https://ctdbase.org/) and GeneCards (https://www.genecards.org/) databases; the screening conditions were set at: CTD: Score_gda ≥ 0.05; GeneCards: Score ≥ 5). As a result, 1333 targets were retrieved on CTD and 751 targets on GeneCards. Overlapping targets from the two databases were screened and redundant targets were eliminated, from which 487 HCC-related targets were obtained.

#### Protein–protein interaction (PPI) network

With the “species” limited to “Homo sapiens”, the interaction network for YFJP compound targets and HCC-related targets was obtained from the STRING database (https://string-db.org).

#### Network construction and analysis

The main types of network diagrams included were HCC-related target, YFJP-HCC-compound-target, YFJP and HCC overlapping target-compound-HCC-YFJP and key target-compound-HCC-YFJP networks. Cytoscape (https://cytoscape.org/, ver. 3.7.1) were used to draw all the above network relationship diagrams and analyze the results; the key targets for YFJP treatment of HCC were screened based on the following criteria: BC ≥ Avg (BC), CC ≥ Avg (CC), and De ≥ Avg (De).

#### Cluster analsis

In large PPI networks, the tightly connected regions that may represent molecular complexes are defined as topological modules or clusters [19], which possess pure network properties. The aggregation of nodes with similar or related functions in the same network is called a functional module. The disease module is a set of network components that collectively disrupt cell functions and then cause a specific disease phenotype. Since the topological module, functional module, and disease module have the same meaning in the network, the functional module is equivalent to the topological module, and the disease module can be regarded as the interference and interruption of the functional module [20]. Through the Cytoscape plug-in MCODE, we performed cluster analysis to obtain the topological modules.

#### Enrichment analysis

Gene ontology (GO) analysis and Kyoto encyclopedia of genes and genomes (KEGG) analysis were performed using the functional enrichment website, DAVID (https://david.ncifcrf.gov/), to investigate the cell functions and signaling pathways that were mainly affected by the key targets for YFJP treatment of HCC [21].

### In vitro validation

#### Cell culture

The HCC line HepG2, which were purchased from Beijing DINGGUO CHANGSHENG Biotechnology Co., Ltd. (Beijing, China), were routinely cultured in DMEM (Gibco, Grand Island, NY, USA) containing 10% FBS (Thermo Fisher Scientific, Rochester, NY, USA). The cells were cultured in a 37 °C incubator with 5% CO_2_ and were used for subsequent experimentation upon attaining a logarithmic growth phase.

#### Preparation of medicated serum

Eighteen male Sprague Dawley rats (weight 200 ± 10 g, 7 weeks old, SPF grade, purchased from SPF (Beijing) Experimental Animal Technology Co., Ltd., Beijing, China) were fed adaptively in the for one week, with free access to food and water in standard animal quarters held at 22 ± 2 ℃, 40–6% humidity, 12 h: 12 h light–dark cycle, and good ventilation. The rats were randomly assigned to one of three groups (n = 6 each), namely the control, the YFJP-L, and the YFJP-H. Concentrated YFJP was prepared as described above. Rats in the YFJP-L group were intragastrically administered with 0.8 ml each time, rats in the YFJP-H group with 1.6 ml each time, and rats in the control group received 0.8 ml normal saline, twice a day, for five days. Before the final gavage, rats were fasted for 12 h with free access to water. At two h after the last administration, pentobarbital sodium (30 mg/kg) was injected intraperitoneally for anesthesia, and blood was collected from abdominal aorta under sterile conditions. The blood was centrifuged at 3000 rpm for 10 min, and serum was passed through a 0.22 μm filter membrane, inactivated at 56 ℃ for 30 min and stored at − 20 ℃ for later use. The serum of the control group was blank serum, and the serum of two YFJP dosage groups was drug containing serum. For use in cell culture, the above serum was mixed with complete culture medium at 1:9 volume ration.

The dose for rats (D2) was calculated as follows: D2 = D1 × 6.25 mg/kg, where D1 represented the human dose. The dose for human adults was 109 g/60 kg/day, thus giving a scaled dose for rats of 11.35 g/kg, which was administered as two daily intragastric feedings of 0.8 ml each.

### Scratch test

Cells in logarithmic growth were seeded in a 24-well plate (3 × 10^4^ cells/well). After the cells had reached a sub-confluency state, a 2-mm scratch was made along the midline of the plate. Cells were assigned into the blank control group (blank control) (cells cultured with normal culture medium), blank serum group (blank serum) (cells cultured with normal culture medium supplemented with blank serum), YFJP low-dose group (YFJP-L) and YFJP high-dose group (YFJP-H), with six replicate wells set up for each group. When the cells were cultured in drug-containing or drug-free medium for 0, 6, and 24 h, the plates were photographed under an inverted microscope (Olympus, Tokyo, Japan), and the cell migration of each group was observed.

#### Transwell assay

The bottom of the Transwell chamber membrane was coated with Matrigel dilution gel and hydrated. Cells in the logarithmic growth phase were prepared into single cell suspension (5 × 10^5^ cells/ml) using serum-free medium. Cells were grouped into the blank control group (blank control), blank serum group (blank serum), YFJP low-dose group (YFJP-L), and YFJP high-dose group (YFJP-H), with six replicate wells set up for each group. Next, 200 μL of single cell suspension was added to the apical chamber of the Transwell chamber. Then, 500 μL of DMEM culture medium containing 10% FBS was added to the basolateral chamber of the blank control group. Blank serum group, YFJP-L group and YFJP-H group were added with 10% blank serum or drug-containing serum for 24 h. After further culture for 24 h, the Transwell chambers were removed, with the cells on the surface of the apical chamber membrane were collected with a cotton swab. The cells were fixed with 4% paraformaldehyde for 30 min, stained with 1% toluidine blue (Sigma-Aldrich Chemical Company, St Louis, MO, USA) at room temperature for 20 min, and finally counted under an inverted microscope (Olympus, Tokyo, Japan).

### 5-ethynyl-2′-deoxyuridine (EdU) assay

Cells in logarithmic growth phase were seeded in a 24-well plate (3 × 10^4^ cells/well) and cultured to a sub-confluency, followed by setting up the blank control group (blank control), blank serum group (blank serum), YFJP low-dose group (YFJP-L), and YFJP high-dose group (YFJP-H), with six replicate wells set up for each group. Following cell culture in drug-containing or drug-free medium for 24 h, EdU was added to the culture solution to a final concentration of 10 µM, followed by further incubation for 2 h. The medium was removed and the cells were then fixed in phosphate buffer saline (PBS) solution containing 4% paraformaldehyde for 15 min at room temperature. Cells were washed twice with PBS containing 3% bovine serum albumin and incubated with PBS containing 0.2% Triton-100 at room temperature for 15 min. After two rinses with PBS containing 3% BSA, 100 μL of the staining solution was added to each well, followed by incubation at room temperature for 30 min in the dark. Subsequently, 4′-6-diamidino-2-phenylindole (DAPI) (Beyotime, Shanghai, China) was used to stain the nuclei for 5 min. The cells were randomly observed in 6—10 fields under a fluorescence microscope (Olympus, Tokyo, Japan), and the number of positive cells in each field was recorded. EdU labeling rate (%) = the number of positive cells / (the number of positive cells + the number of negative cells) × 100%.

### TUNEL staining

Cell apoptosis was detected using a TUNEL detection kit (Roche, Indianapolis, IN, USA) following the manufacturer’s protocols. Cells in logarithmic growth phase were seeded in a 24-well plate (3 × 10^4^ cells/well) and cultured to sub-confluency. The blank control group (blank control), blank serum group (blank serum), YFJP low-dose group (YFJP-L), and YFJP high-dose group (YFJP-H) were established, with six replicate wells set up for each group. Cells were cultured for 24 h in drug-containing or drug-free medium, after which they were and immersed three times in PBS (three min each time), and fixed in 4% paraformaldehyde for 20 min. Next, the cells were infiltrated with 0.2% Triton X-100 for 15 min at room temperature, followed by incubation with 80 μL of TUNEL reaction solution in a wet box in the dark at 37 °C for 90 min. Finally, nuclei of the cells were stained with 10 μg/ml DAPI (Beyotime, Shanghai, China). After staining, the cells were observed and photographed under a fluorescent microscope (Olympus, Tokyo, Japan). TUNEL-positive apoptotic cells showed green nuclear stain, and the total number of all cells was counted using DAPI blue nuclear staining. The apoptotic rate was the percentage of apoptotic positive cells to the total number of cells.

### RNA extraction and quantitative reverse transcription-polymerase chain reaction (qRT-PCR)

Cells in logarithmic growth phase were seeded in a 24-well plate (3 × 10^4^ cells/well) and cultured to sub-confluency. The cells were assigned into the blank control group (blank control), blank serum group (blank serum), YFJP low-dose group (YFJP-L), and YFJP high-dose group (YFJP-H), with six duplicate wells set up for each group. Cells were cultured in drug-containing or drug-free medium for 24 h. Next, total RNA was extracted using Trizol reagent (15596026, Invitrogen, Carlsbad, California, USA), and the RNA was reverse-transcribed into complementary DNA (cDNA) according to the instructions of the PrimeScript RT reagent kit (RR047A, Takara Bio Inc., Otsu, Shiga, Japan). The synthesized cDNA was detected using a Fast SYBR Green PCR kit (Applied Biosystems, Carlsbad, CA, USA) and the ABI PRISM 7300 qRT-PCR system (Applied Biosystems, Carlsbad, CA, USA). With glyceraldehyde-3-phosphate dehydrogenase (GAPDH) as the internal reference, the relative gene expression was analyzed by means of the 2^−ΔΔCt^ method. The primer sequences are shown in Additional file 1: Table S1.

### Statistical analysis

Statistical analysis of the data in this study was performed using the statistical software SPSS 21.0 (IBM Corp., Armonk, NY, USA). Measurement data were expressed as mean ± standard deviation. First, a test of normality and homogeneity of variance was performed. Data between two groups obeying normal distribution and homogeneous variance were compared employing *t*-tests, and comparisons between multiple groups were conducted using one-way analysis of variance (ANOVA), followed by Tukey’s post hoc test. Comparisons among multiple groups at different time points were carried out using repeated measures ANOVA, followed by Tukey’s post hoc test. The Kaplan–Meier method was applied to calculate the patients’ survival curve, and log-rank was used for univariate analysis of survival rates between groups. Survival time was expressed as a median (75% quantile). Factors affecting the prognosis obtained from the univariate analysis were introduced into a COX proportional hazard model for multivariate analysis, followed by observation of these factors. *p* < 0.05 indicated that the difference was statistically significant.

## Results

### Univariate and multivariate analyses

There were 477 patients enrolled in this study, one third of whom received treatment with YFJP. Baseline characteristics of the patients are shown in Table 1. Clinical data analysis displayed that drinking alcohol (adjusted HR = 1.53, 95%CI 0.76–1.59, *p* = 0.009), baseline PVTT (adjusted HR = 1.45, 95%CI 1.08–1.95, *p* = 0.011), AFP ≥ 400 ng/l (adjusted HR = 1.77, 95%CI 1.55–2.02, *p* = 0.003), total bilirubin level (TBIL) ≥ 18.8 μM (adjusted HR = 1.41, 95%CI 1.01–1.96, *p* = 0.042) were independent risk factors for poor prognosis, while red blood cell (RBC) density ≥ 4 × 10^9^/l (adjusted HR = 0.63, 95%CI 0.42–0.93, *p* = 0.023) and TCM treatment (adjusted HR = 0.48, 95%CI 0.38–0.60, *p* < 0.001) were independent protective factors for more favorable prognosis (Table 2).

**Table 1:** Patients’ baseline characteristics between the YFJP group and the non-YFJP group

t Variable	YFJP (n = 159)	non-YFJP (n = 318)	*p* value
Age (< 50/ ≥ 50 years)	32/127	75/242	0.393
Gender (male/female)	122/37	245/73	0.939
Tumor numbe (Single/Multiple)	77/82	150/168	0.795
Maximal tumor size (≤ 5/ > 5 cm)	33/126	150/168	< 0.001
Liver cirrhosis (Yes/No)	148/11	301/17	0.491
WBC (≤ 4 × 10^9^/l/ > 4 × 10^9^/l)	85/74	201/117	0.041
RBC (≤ 4 × 10^9^/l/ > 4 × 10^9^/l)	70/89	119/199	0.165
Platelet (≤ 100 × 10^9^/l/ > 100 × 10^9^/l)	54/105	139/179	0.041
Serum AFP levels (≥ 400/ < 400 ng/ml)	50/109	127/191	0.07
TBIL (≥ 18.8 / < 18.8 μmol/l)	81/78	216/102	< 0.001
ALB (≥ 40 / < 40 g/l)	38/121	56/262	0.104
ALT (≥ 50 / < 50 U/l)	40/119	113/205	0.022
AST (≥ 40 / < 40 U/l)	72/87	220/98	< 0.001
Child stage (Grade A/Grade B/Grade C)	75/59/25	108/133/77	0.011
NLR (≤ 2.4/ > 2.4)	66/93	119/199	0.388
BCLC stage (A/B/C/D)	81/40/12/25	53/93/95/77	< 0.001
Smoking (Yes/No)	75/84	110/208	0.008
Drinking (Yes/No)	73/86	114/204	0.034
Diabetes (Yes/No)	35/124	71/247	0.938
Hypertension (Yes/No)	52/107	76/242	0.041
γ-GGT (> 60/60 U/l)	61/98	206/112	< 0.001

*YFJP* Yangyin Fuzheng Jiedu, *AFP* alphafetoprotein, *TBIL* total body irradiation, *ALB* albumin, *ALT* alanine aminotransferase, *AST* aspartate aminotransferase, *NLR* neutrophil-to-lymphocyte ratio, *TNM* tumor-node-metastasis, *BCLC* Barcelona clinic liver cancer, *WBC* white blood cell, *RBC* red blood cell

**Table 2:** Univariate and multivariate analyses of variables influencing survival of 477 patients with HCC

Characteristics		Univariate analysis		Multivariate analysis	
		Exp(*β*)(95% CI)	*p* value	Exp(*β*)(95% CI)	*p* value
Age (years)	< 50	0.724 (0.559–0.936)	0.014		
	≥ 50				
Gender	male	0.747 (0.576–0.968)	0.027	–	–
	female				
Tumor number	Single	1.727 (1.398–2.133)	< 0.001	–	–
	Multiple				
Maximal tumor size	> 5 cm	2.420 (1.954–2.997)	< 0.001	–	–
	≤ 5 cm				
Liver cirrhosis	Yes	0.998 (0.546–1.823)	0.995	–	–
	No				
AFP	> 400 ng/ml	2.672 (2.135–3.344)	< 0.001	1.536 (1.150–2.051)	0.004
	≤ 400 ng/ml				
TBIL	> 18.8 μmol/l	1.497 (1.198–1.870)	< 0.001	–	–
	≤ 18.8 μmol/l				
ALB	> 40 g/l	0.764 (0.566–1.031)	0.078	–	–
	≤ 40 g/l				
ALT	> 50 U/l	1.234 (0.990–1.538)	0.061	–	–
	≤ 50 U/l				
AST	> 40 U/l	1.720 (1.367–2.165)	< 0.001	–	–
	≤ 40 U/l				
	A	1.259 (1.097–1.445)	0.001	–	–
Child stage	B				
	C				
NLR	≤ 2.4	1.605 (1.291–1.996)	< 0.001	–	–
	> 2.4				
BCLC grade	A–B	1.376 (1.261–1.501)	< 0.001	–	–
	C–D				
Smoking	Yes	1.134 (0.916–1.403)	0.249	–	–
	No				
Drinking	Yes	1.330 (1.075–1.645)	0.009	1.534 (1.112–2.117)	0.001
	No				
Diabetes	Yes	0.877 (0.678–1.134)	0.318	–	–
	No				
Hypertension	Yes	0.810 (0.638–1.028)	0.084	–	–
	No				
TCM treatment	Yes	0.282 (0.218–0.366)	< 0.001	0.480 (0.380–0.600)	< 0.001
	No				
PVTT	Yes	1.392 (1.125–1.721)	0.002	1.459 (1.089–1.955)	0.011
	No				
RBC	> 4 × 10^9^/l	0.868 (0.696–1.083)	0.21	0.633 (0.427–0.939)	0.023
	≤ 4 × 10^9^/l				

*CI* confidence interval, *YFJP* Yangyin Fuzheng Jiedu, *AFP* alphafetoprotein, *TBIL* total body irradiation, *ALB* albumin, *ALT* alanine aminotransferase, *AST* aspartate aminotransferase, *NLR* neutrophil-to-lymphocyte ratio, *TNM* tumor-node-metastasis, *BCLC* Barcelona clinic liver cancer, *PVTT* portal vein tumor thrombus, *RBC* red blood cell

### Survival analysis

**Fig. 1:**
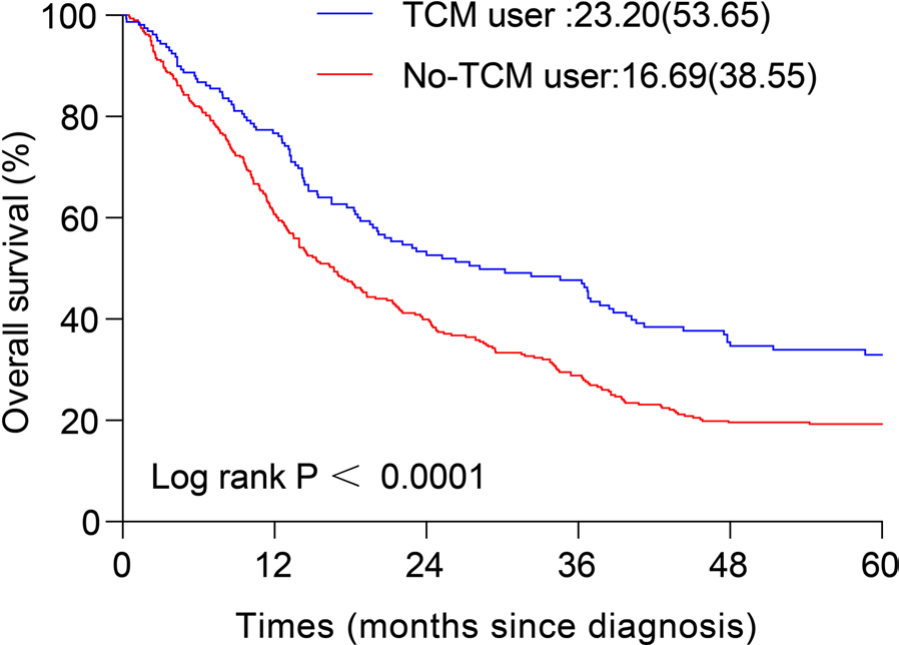
Kaplan–Meier analysis. Kaplan–Meier analysis for the 5-year cumulative survival rate between patients in the YFJP group (n = 159) and non-YFJP group (n = 318)

The survival curves of HCC patients in the YFJP and non-YFJP groups are shown in Fig. 1. The analyses revealed that the 5-year cumulative survival rate in the YFJP group was significantly higher than those in the non-YFJP group (survival time of the YFJP group: 23.20 (53.65) *vs*. the non-YFJP group: 16.69 (38.55); *p* < 0.0001).

### YFJP identification and content determination

Main components of YFJP were identified and analyzed by HPLC analysis with UV detection (Additional file 2: Figure S1). By comparing the retention time of the standard control compound, two known ingredients in YFJP were identified. The relative contents of isoflavone glucoside and paeonol in YFJP were 31.84% and 40.55%, respectively.

### HCC-related target network

**Fig. 2:**
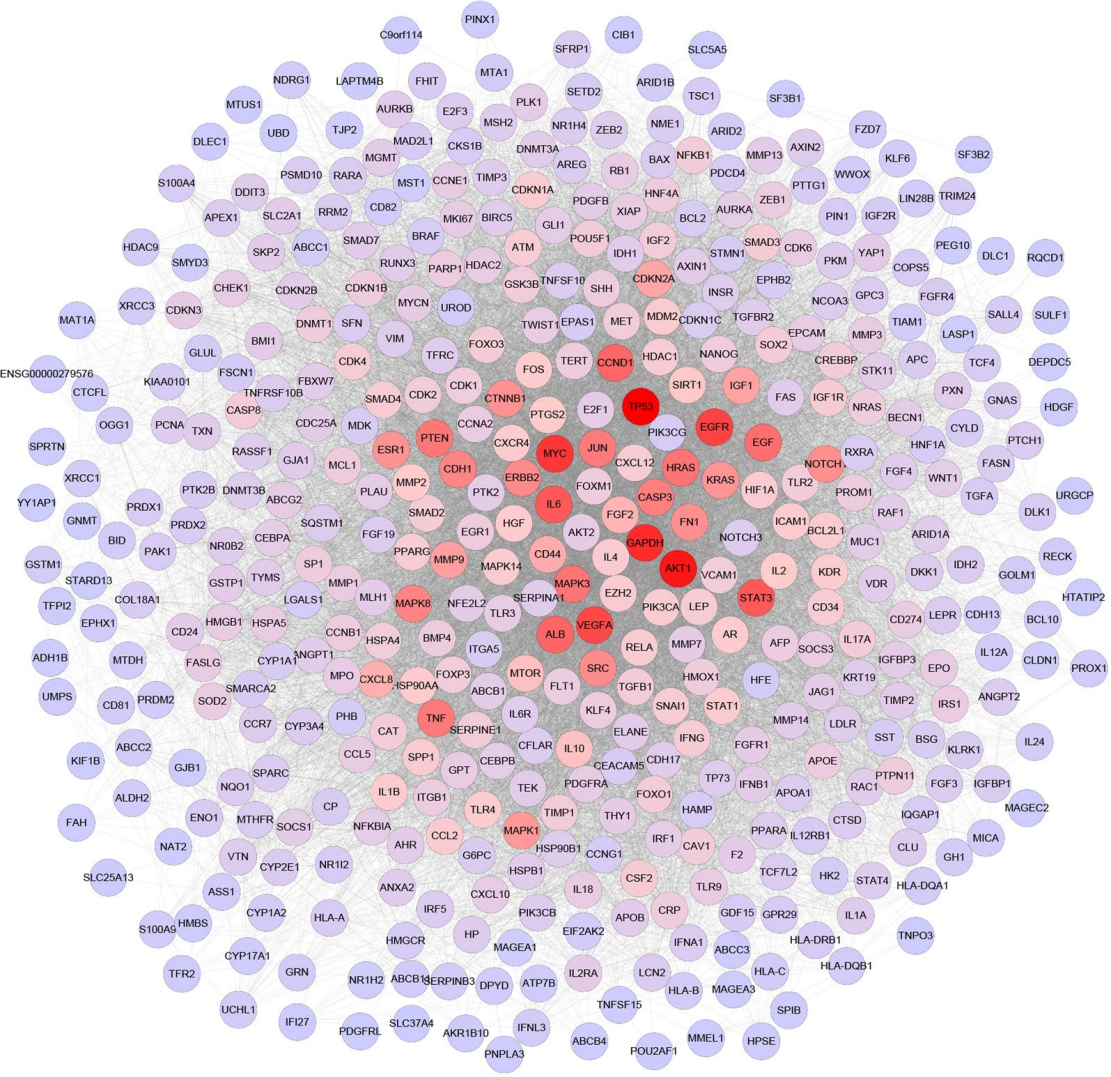
The HCC-related target network

The association information among 487 HCC-related genes was obtained from the PPI. A gene–gene interaction network was then constructed. The network had a total of 451 nodes (36 target genes were not present in “Homo sapiens” and thus were deleted) and 15,829 edges (Fig. 2). A total of ten red nodes (TP53, AKT1, GAPDH, MYC, EGFR, VEGFA, STAT3, IL-6, ALB, and CCND1) had high degree values, and each node had a relatively large number of edges (329 for TP53, 308 for AKT1, 308 for GAPDH, 281 for MYC, and 270 for EGFR, 265 for VEGFA, 250 for STAT3, 250 for IL-6, 239 for ALB, and 238 for CCND1). The above results suggest that the above ten genes may be key genes for HCC occurrence.

### Cluster analysis for HCC-related targets

**Fig. 3:**
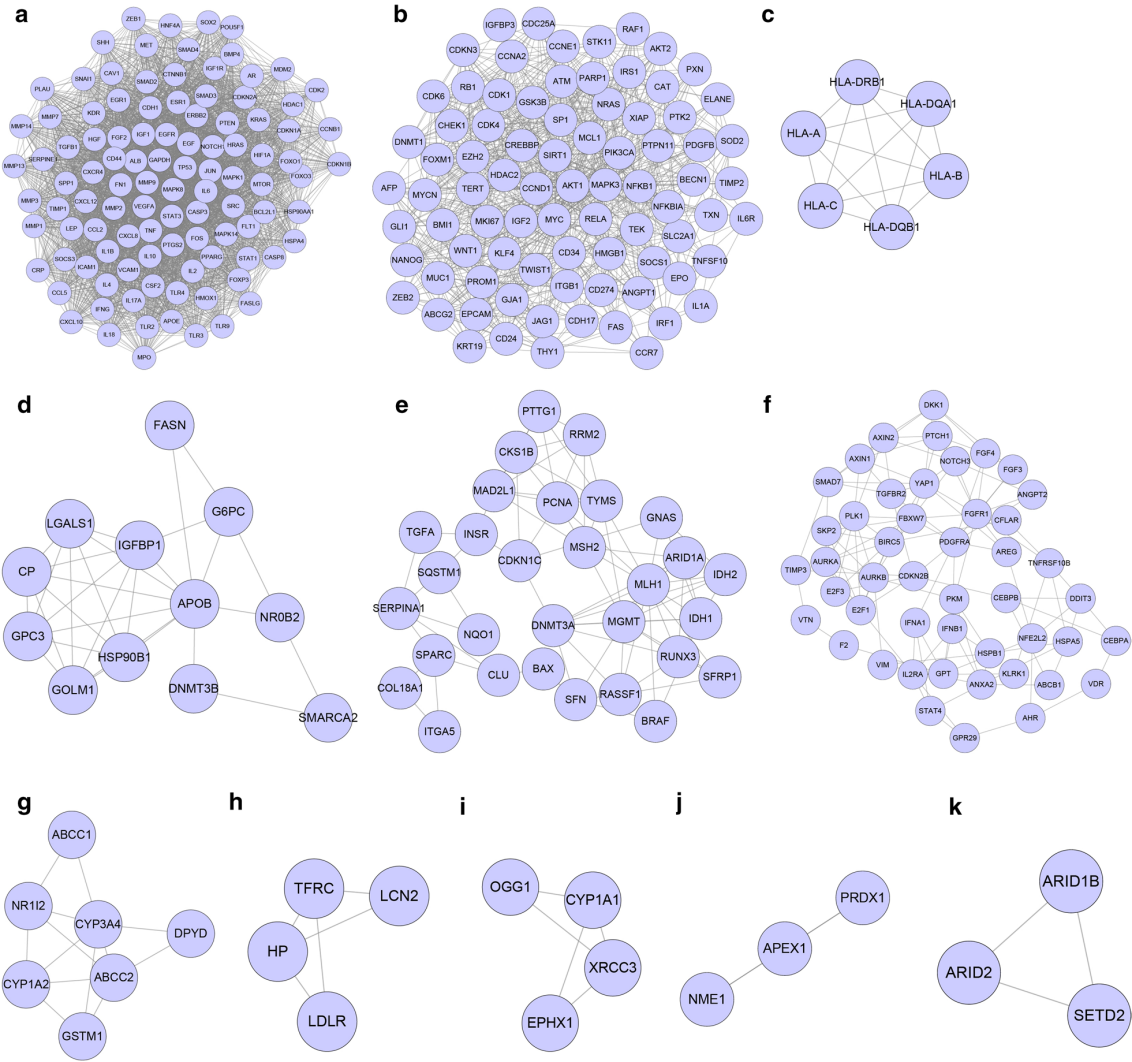
Cluster analysis for HCC-related targets

**Fig. 4:**
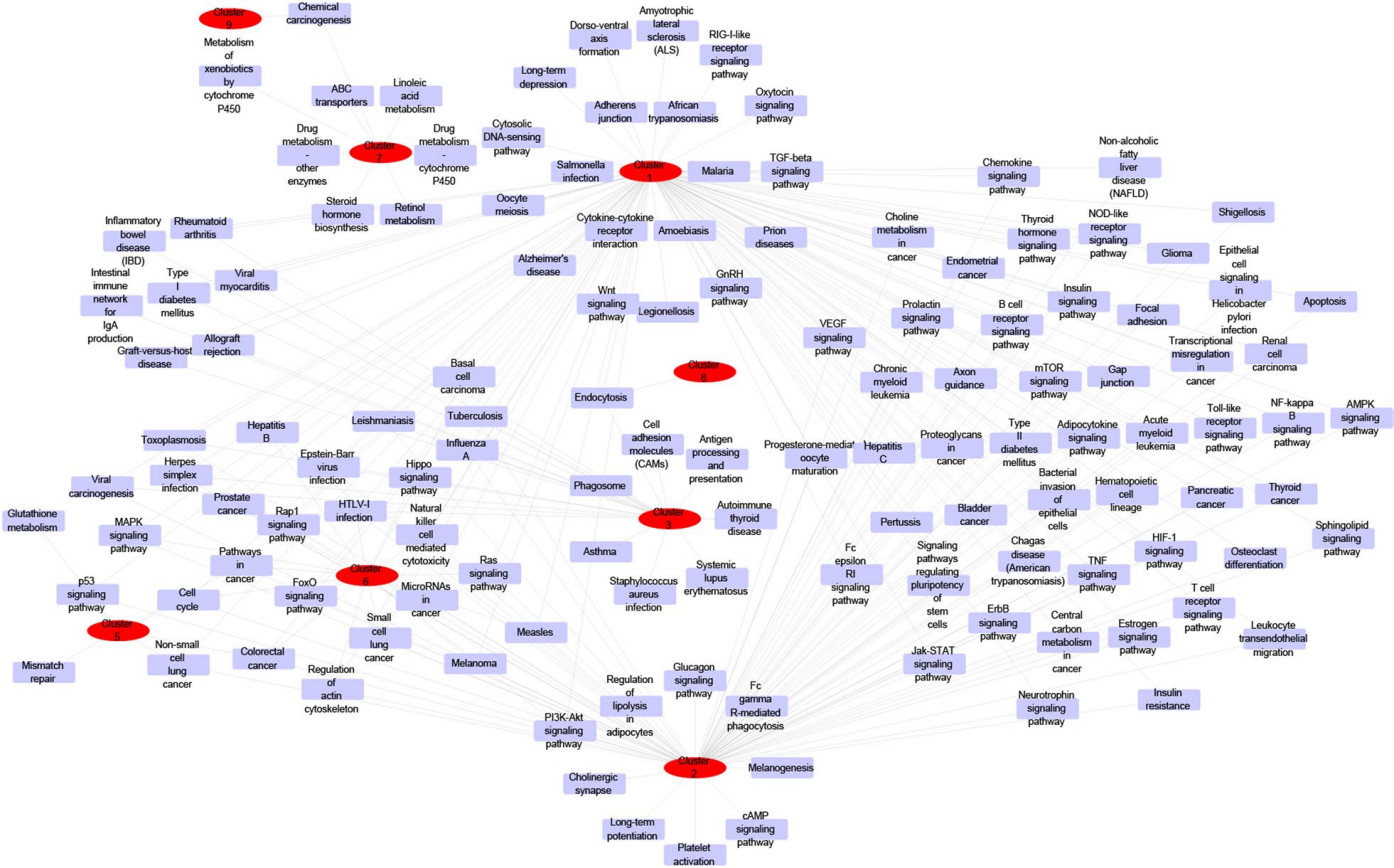
The network for KEGG enrichment analysis

Cluster analysis was performed on the HCC-related target network, and a total of 11 topological modules were obtained (Additional file 1: Table S2, Fig. 3). They were: Cluster 1: score = 72.039, Nodes = 104, Edges = 3710 (Fig. 3a); Cluster 2: score = 24.42, Nodes = 82, Edges = 989 (Fig. 3b); Cluster 3: score = 6, Nodes = 6, Edges = 15 (Fig. 3c); Cluster 4: score = 5.455, Nodes = 12, Edges = 30 (Fig. 3d); Cluster 5: score = 5.448, Nodes = 30, Edges = 79 (Fig. 3e); Cluster 6: score = 5.348, Nodes = 47, Edges = 123 (Fig. 3f); Cluster 7: score = 4.333, Nodes = 7, Edges = 13 (Fig. 3g); Cluster 8: score = 3.333, Nodes = 4, Edges = 5 (Fig. 3h); Cluster 9: score = 3.333, Nodes = 4, Edges = 5 (Fig. 3i); Cluster 10: score = 3, Nodes = 3, Edges = 3 (Fig. 3j); Cluster 11: score = 3, Nodes = 3, Edges = 3 (Fig. 3k). KEGG analysis was performed on each Cluster, and the results are illustrated in Fig. 4. There were 106 types of signal pathways with significant difference (*p* < 0.05) in Cluster 1, 85 types in Cluster 2, 23 types in Cluster 3, 7 types in Cluster 5, 19 types in Cluster 6, 8 types in Cluster 7, 1 type in Cluster 8, and 2 types in Cluster 9.

### YFJP-TCM-compound-target network and cluster analysis

**Fig. 5:**
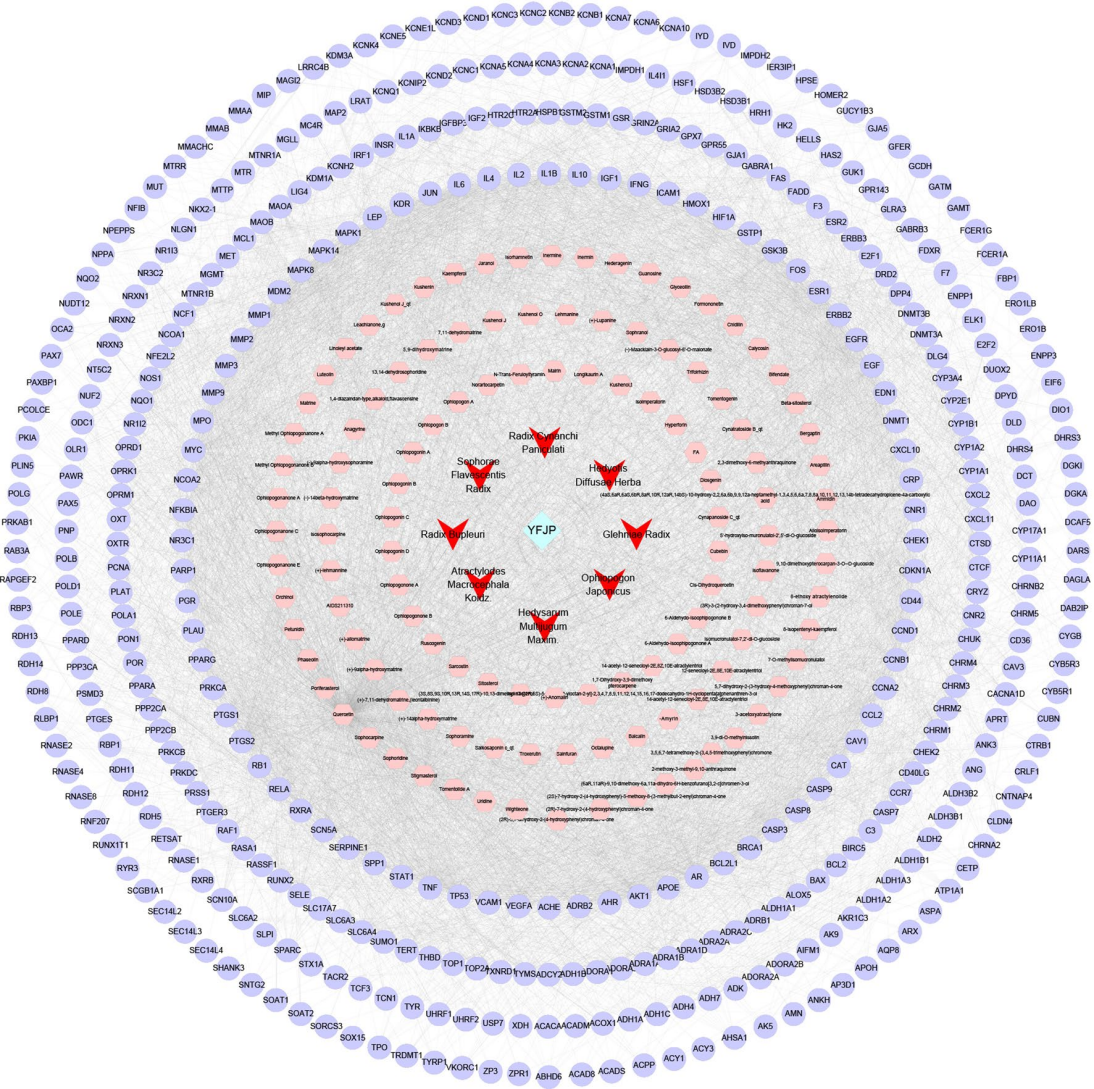
The YFJP-TCM-compound-target network and cluster analysis. The YFJP-TCM-compound-target network contains 1 TCM prescription, 8 TCMs, 120 compounds and 411 targets (The blue diamond node represents the TCM prescription, the red V-shaped nodes represent TCMs, the pink hexagons represent specific compounds, and the purple circles represent targets)

The YFJP-TCM-compound-target network consisted of 540 nodes and 7577 edges (Fig. 5a), which included 1 TCM formula, eight Chinese medicines, 120 compounds, and 411 targets. In this network, many targets can be regulated by multiple compounds at the same time (inner nodes, such as PTGS2, PTGS1, NCOA2, SCN5A, RXRA, PRSS1, ADRB2, PPARG, GABRA1, AR, DPP4, ESR1, CHRM1, ADRA1B and PGR, etc.), but there were 204 targets which can only be regulated by one compound (outer node, such as HRH1, HPSE, IER3IP1, CD44, MDM2, PCNA, CASP7, MCL1, PTGES, NUF2, ADCY2, TYMS, NT5C2, ADK, and IMPDH1). For example, PTGS2 is regulated by all components of TCMs except *Ophiopogon japonicus (Thunb.) Ker Gawl.*, and ESR1 is regulated by all compounds in TCMs except *Glehnia littoralis F.Schmidt ex Miq.* and *Atractylis macrocephala (Koidz.) Hand.-Mazz*. The aforementioned results suggest that the compounds in YFJP may act synergistically on these targets, thus exerting pharmacological effects to inhibit HCC. In addition, the multi-component, multi-target, and multi-disease treatment characteristics of TCM prescriptions are also implied.

### Potential target-compound-TCM-YFJP network and cluster analysis

**Fig. 6:**
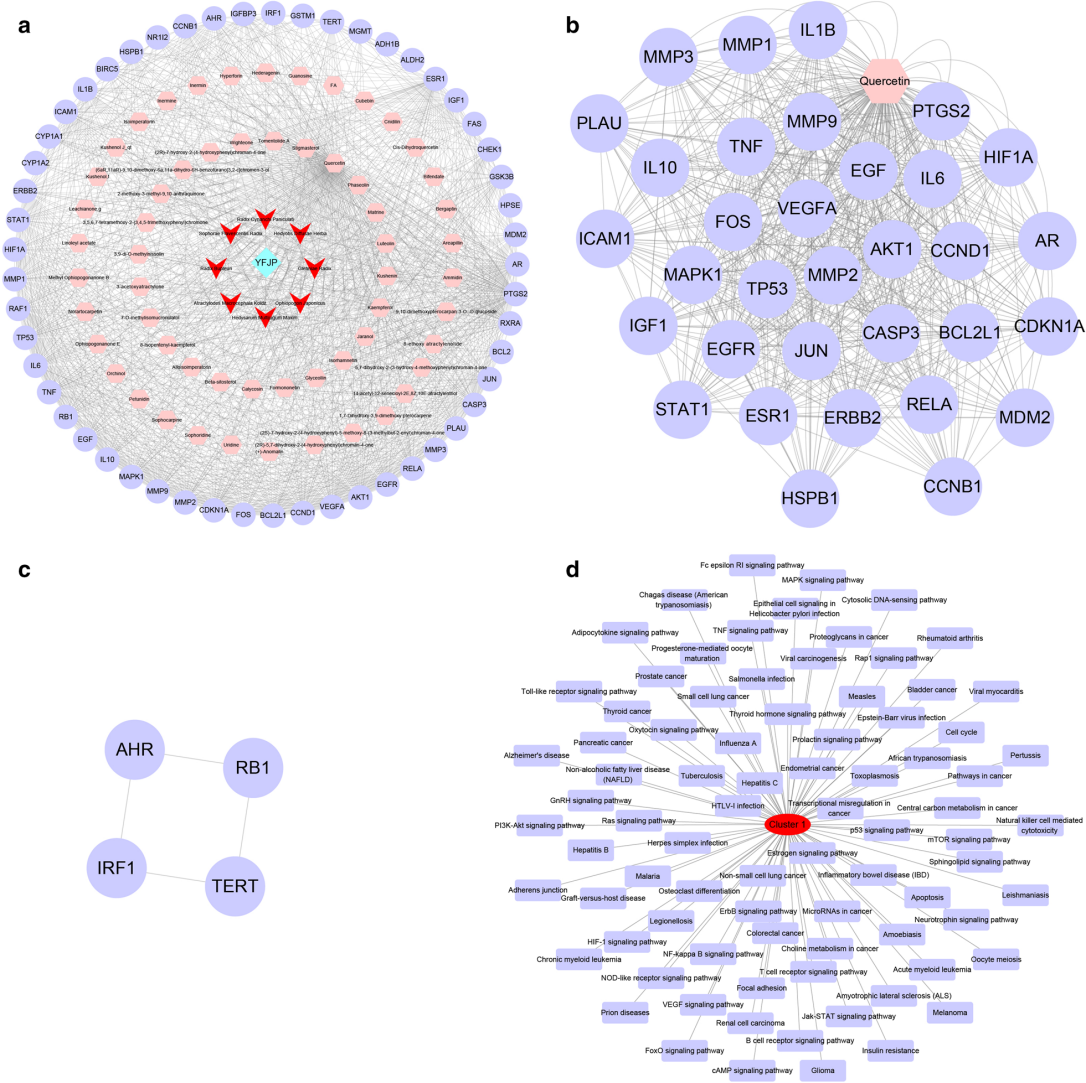
The potential target-compound-HCC-YFJP network and cluster analysis. **a** The potential target-compound-HCC-YFJP network contains 1 TCM prescription, 8 TCMs, 58 compounds, and 53 targets (The blue diamond node represents TCM prescription, the red V-shaped nodes represent TCM, the pink hexagons represent compounds, and the purple circles represent targets). **b**, **c** Cluster analysis for topological modules. **d** KEGG analysis network for Cluster 1

The overlapping targets of YFJP and HCC (potential targets for YFJP treatment of HCC) were screened to obtain the potential target-compound-TCM-YFJP network (Fig. 6a). This network contained 120 nodes and 1214 edges, including 1 Chinese medicine prescription, 8 Chinese medicines, 58 compounds and 53 targets. Cluster analysis was performed on this network, and a total of 2 topological modules were obtained (Fig. 6b, c): Cluster 1: score = 29.879, Nodes = 34, Edges = 613 (Fig. 6b); Cluster 2: score = 2.677, Nodes = 4, Edges = 4 (Fig. 6c). Cluster 1 included 1 compound Quercetin and 33 targets (BCL2L1, CCND1, VEGFA, AKT1, EGFR, RELA, CCNB1, MMP3, HSPB1, PLAU, IL1B, CASP3, ICAM1, JUN, ERBB2, STAT1, HIF1A, MMP1, PTGS2, AR, TP53, IL6, TNF, EGF, IL10, MAPK1, IGF1, MMP9, ESR1, MMP2, MDM2, CDKN1A, and FOS). Cluster 1 included 4 targets (Additional file 1: Table S3). KEGG analysis was performed on each topological module, and the results are shown in Fig. 6. There were 83 signal pathways with significant difference (*p* < 0.05) in Cluster 1 (Fig. 6d).

### Key target-compound-TCM-YFJP network and cluster analysis

**Fig. 7:**
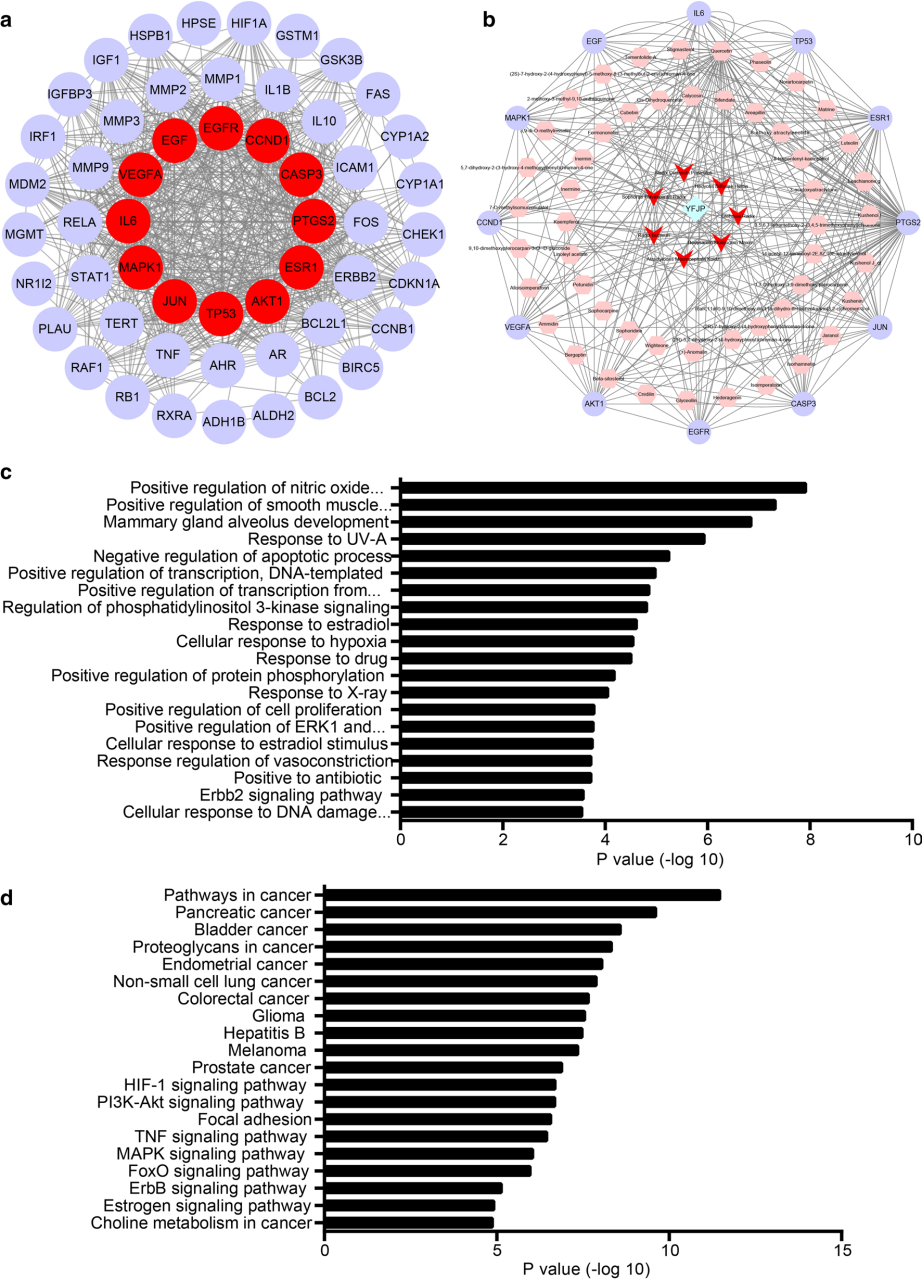
The key target-compound-HCC-YFJP network and cluster analysis. **a** Network for putative targets for YFJP treatment of HCC. **b** The key target-compound-HCC-YFJP network includes 1 TCM prescription, 7 Chinese medicines, 51 compounds and 12 targets (The blue diamond node represents TCM prescription, the red V-shaped nodes represent TCM, the pink hexagons represent compounds, and the purple circles represent targets). **c** GO analysis for key targets. **d** KEGG analysis for key targets

Putative targets for YFJP treatment of HCC were screened, from which 12 key targets (TP53, EGFR, EGF, CCND1, JUN, AKT1, IL6, MAPK1, VEGFA, ESR1, CASP3, and PTGS2) were obtained (Fig. 7a). Subsequently, a key target-compound-TCM-YFJP network was delineated (Fig. 7b). This network contained 71 nodes and 287 edges, including 1 TCM prescription, 7 TCMs, 51 compounds and 12 key targets. Enrichment analysis was then performed on these 12 targets. As displayed in the GO analysis results (Fig. 7c), 103 cell functions were significantly affected (*p* < 0.05), including (GO: 0045429) positive regulation of nitric oxide biosynthetic process, (GO: 0048661) positive regulation of smooth muscle cell proliferation, (GO: 0060749) mammary gland alveolus development, (GO: 0,070,141) response to UV-A, (GO: 0,043,066) negative regulation of apoptotic process, (GO: 0,045,893) positive regulation of transcription, DNA-templated, (GO: 0045944) positive regulation of transcription from RNA polymerase II promoter, (GO: 0014066) regulation of phosphatidylinositol 3-kinase signaling, (GO: 0032355) response to estradiol, (GO: 0071456) cellular response to hypoxia, (GO: 0042493) response to drug, (GO: 0001934) positive regulation of protein phosphorylation, (GO: 0010165) response to X-ray, (GO: 0008284) positive regulation of cell proliferation, (GO: 0070374) positive regulation of ERK1 and ERK2 cascade, (GO: 0071392) cellular response to estradiol stimulus, (GO: 0045907) positive regulation of vasoconstriction, (GO: 0046677) response to antibiotic, (GO: 0038128) ERBB2 signaling pathway, (GO: 0006974) cellular response to DNA damage stimulus. As revealed by the KEGG analysis (Fig. 7d), 65 types of signaling pathway were significantly affected (*p* < 0.05), including (hsa05200) pathways in cancer, (hsa05212) pancreatic cancer, (hsa05219) bladder cancer, (hsa05205) proteoglycans in cancer, (hsa05213) endometrial cancer, (hsa05223) non-small cell lung cancer, (hsa05210) colorectal cancer, (hsa05214) glioma, (hsa05161) hepatitis B, (hsa05218) melanoma, (hsa05215) prostate cancer, (hsa04066) HIF-1 signaling pathway, (hsa04151) PI3K-Akt signaling pathway, (hsa04510) focal adhesion, (hsa04668) TNF signaling pathway, (hsa04010) MAPK signaling pathway, (hsa04068) FoxO signaling pathway, (hsa04012) ErbB signaling pathway, (hsa04915) Estrogen signaling pathway and (hsa05231) choline metabolism in cancer.

### YFJP inhibits migration and invasion of HepG2 cells

**Fig. 8:**
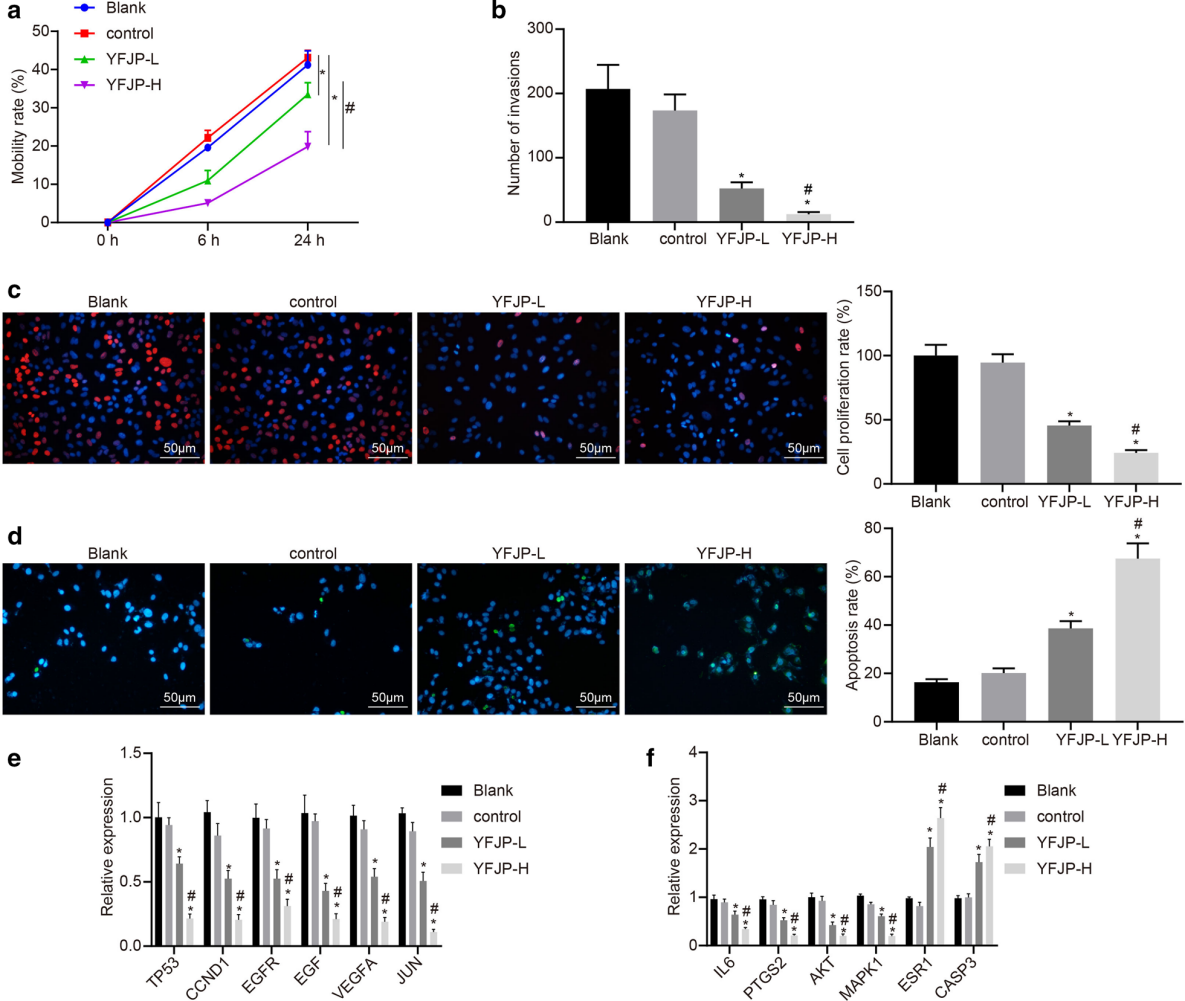
YFJP inhibits the migration, invasion, and proliferation, while inducing the apoptosis of HepG2 cells. **a** The migration of HepG2 cells as detected by scratch test (× 200). **b** The invasion of HepG2 cells as detected by Transwell assay (×200). **c** The proliferation of HepG2 cells as detected by EdU assay. **d** The apoptosis of HepG2 cells as detected by TUNEL staining (×200). **e**, **f** The mRNA expression as detected by qRT-PCR. * *p* < 0.05 *vs*. the blank control group. ^#^ *p* < 0.05 *vs*. the YFJP-L group. These data were measurement data, expressed as mean ± standard deviation. Data among multiple groups were compared using one-way ANOVA, followed by Tukey’s post hoc test. The experiments were repeated three times

The effect of YFJP on migration of HepG2 cells is displayed in Fig. 8a. Compared with the blank control group, there was no significant difference in the migration of HepG2 cells in the blank serum group, while the YFJP-L (*p* < 0.05) and YFJP-H groups (*p* < 0.05 or *p* < 0.01) showed notably reduced migration of HepG2 cells. This experimental result revealed that YFJP could inhibit migration of HepG2 cells in a time- and dose-dependent manner. Furthermore, the results from the Transwell assay (Fig. 8b) showed no significant difference in the number of invasive HepG2 cells between the blank control group and the blank serum group. However, compared with the blank control group, the cell invasion was significantly reduced in the YFJP-L (*p* < 0.05) and YFJP-H groups (*p* < 0.01). Overall, YFJP could inhibit the migration and invasion ability of HepG2 cells.

### YFJP inhibits proliferation and induces apoptosis of HepG2 cells

EdU results (Fig. 8c) showed no marked difference in cell proliferation in the blank serum group compared with the blank control group. Relative to the blank control group, the cell proliferation rate in the YFJP-L group (*p* < 0.05) and the YFJP-H group (*p* < 0.01) was significantly reduced. Based on the results from TUNEL staining (Fig. 8d), there was no marked difference in the number of apoptotic cells in the blank serum group in comparison with the blank control group. However, the number of apoptotic cells in the YFJP-L group (*p* < 0.05) and the YFJP-H group (*p* < 0.01) was notably increased compared to the blank control group. The above experimental results demonstrated that YFJP could induce HepG2 cell apoptosis in a dose-dependent manner. The PCR results (Fig. 8E, F) showed that YFJP significantly inhibited the expression of TP53, CCND1, EGFR, EGF, VEGFA, JUN, IL6, PTGS2, AKT1, and MAPK1 while increasing that of ESR1 and CASP3.

## Discussion

As knowledge about the molecular mechanisms behind tumorigenesis in the liver increases, the development and evaluation of molecularly targeted TCMs may offer a promising perspective for HCC, which remains resistant to treatment, especially in the metastatic phase [22, 23]. TCMs contain multiple components, which have many targets, and network pharmacology analysis combined with experimental assays holds the potential to delineate the underlying possible therapeutic mechanisms [24]. For instance, a recent study examined the molecular mechanisms of Huanglian Jiedu Decoction as an adjunctive therapy in halting the migratory and invasive properties of HCC cells through a network pharmacology analysis combined with experimental models [25]. In the present study, we clarified that YFJP attenuated HCC cell migration and invasion, and induced HCC cell apoptosis through modulating an array of HCC-related genes (including TP53, EGFR, EGF, CCND1, JUN, AKT1, IL6, MAPK1, VEGFA, ESR1, CASP3, and PTGS2), thereby attenuating the aggressiveness of HCC.

The experimental observations of our study unveiled that drinking alcohol, AFP (≥ 400 ng/l), baseline PVTT and TBIL (≥ 18.8 μM) were independent risk factors contributing to poor prognosis of HCC patients, RBC (≥ 4 × 10^9^/l) and TCM treatment were independent protective factors for favorable prognosis. Further survival analyses indicated that HCC patients treated with YFJP exhibited better 5-year cumulative survival rate. The YFJP applied in this study is a Chinese herbal recipe for nourishing yin, supporting the body and removing toxic substances from the body. It consists of eight kinds of plants (*Glehnia littoralis F.Schmidt ex Miq., Hedysarum multijugum Maxim, Atractylis macrocephala (Koidz.) Hand.-Mazz, Bupleurum chinensis DC., Sophora flavescens Aiton, Cynanchum paniculatum (Bunge) Kitagawa, and Hedyotis diffusa Willd.*). Accumulating evidence has identified the clinical therapeutic effect of certain TCMs to improve prognosis and prolong patient survival in the treatment of human cancers. For example, the efficacy of Yiqi Yangyin Jiedu Decoction in advanced lung cancer has been explored in a previous study, which proved to alleviate the qi-yin deficiency syndrome and improve quality of life [26]. This study provided an explanation for the decoction’s efficacy in terms of augmenting T-cell activity and killer T-cell function, but failed to characterize the specific molecular mechanism. Another study investigated another kind of TCM, Fuzheng Kang-Ai Decoction (indicated for supporting the body and combating cancer) as an adjunct treatment option in lung cancer, where the anti-cancer effect of this decoction was ascribed to suppression of tumor cell growth via Akt-dependent repression of p65 and downregulation of MUC1 [27].

In this study, we next proceeded to identify the key target genes associated with the therapeutic effect of YFJP. The initial database-based analysis predicted 12 potential targets (TP53, EGFR, EGF, CCND1, JUN, AKT1, IL6, MAPK1, VEGFA, ESR1, CASP3 and PTGS2) for the antineoplastic properties of YFJP. Of note, the experimental data of our study unveiled that YFJP had the ability to repress the expression and function of such oncogenes as TP53, CCND1, EGFR, EGF, VEGFA, JUN, IL6, PTGS2, AKT1, MAPK1. A previous study has documented the upregulation of TP53 in HCC cell lines, which was shown to stimulate HCC cell viability and migration along with impeding apoptosis [28]. Increasing numbers of studies have suggested that EGF is a critical promoter in the metastasis of primary tumors, which holds the capacity of accelerating tumorigenic and antiangiogenic potential of HCC cells [29]. High expression of the EGF receptor (EGFR) (is positively associated with the proliferative and migratory potential of HCC cells [30]. Furthermore, Vascular Endothelial Growth Factor A (VEGFA) is also an inducer of angiogenesis and tumor necrosis in HCC, and the silencing of VEGFA has been proposed as a therapeutic strategy for HCC [31]. In addition, the high expression of the CCND1 gene, which encodes cyclin D1 protein has been verified in vitro and in vivo to cause appreciable elevation in liver cancer stem cell differentiation by intensifying cell autophagy [32]. JUN has been highlighted for its involvement in modulating HCC cell growth, cell cycle entry and mitosis [33]. The oncogenic cytokine IL6 was found to expedite carcinogenesis and epithelial-mesenchymal transition in HCC [34]. A tumor-supporting role has also been suggested for Prostaglandin-Endoperoxide Synthase 2 (PTGS2) in HCC and colorectal cancer, which plays a critical role in the PI3K/AKT signaling pathway [35, 36]. The identification of tumor-initiating activities of AKT1 inspired the application of an AKT1 inhibitor to stimulate autophagy-associated HCC cell death [37]. Similarly, the MAPK1 gene is also an oncogene leading to accelerated HCC cell proliferation, migration and invasion [38]. At the same time, YFJP was also found to enhance tumor-suppressive genes ESR1 and CASP3. The antineoplastic activity of ESR1 has been also highlighted in a study of Chen et al., which validated its ability in impeding HCC cell growth and triggering cell apoptosis [39]. Besides, the malignancy of HCC cells could be halted by overexpression of CASP3, which could also improve the survival of HCC patients [40]. Corroborating findings have been identified in our study, which demonstrated that YFJP augmented HCC cell migration and invasion, and induced cell apoptosis through regulating these target genes.

## Conclusions

Based on the results observed during this study, we conclude that YFJP attenuated HCC progression through mediating HCC-related genes (including TP53, EGFR, EGF, CCND1, JUN, AKT1, IL6, MAPK1, VEGFA, ESR1, CASP3, and PTGS2). Thus, we are convinced that these results support the implementation of therapeutic directed towards the upregulation of tumor-suppressive genes and downregulation of oncogenes during the application of YFJP. This approach has the potential to serve as a clinically viable method for enhancing the efficacy of YFJP in the treatment of HCC. However, the translation of present finding to human disease will require further research.

## Supplementary information

**Additional file 1.** Additional tables.

**Additional file 2: Figure S1. **HPLC analysis of YFJP. A: Standard control mixture; B: UV tracer of HPLC analysis of YFJP**.**

## Data Availability

The datasets generated and/or analyzed during the current study are available from the corresponding author on reasonable request.

## Abbreviations

YFJP: Yangyin Fuzheng Jiedu Prescription
HCC: Hepatocellular carcinoma
TP53: Tumor protein p53
IL-6: Interleukin 6
EGFR: Epidermal growth factor receptor
ESR1: Estrogen receptor 1
PTGS2: Prostaglandin-endoperoxide synthase 2
EGF: Epidermal growth factor
AKT1: AKT serine/threonine kinase 1
MAPK1: Mitogen-activated protein kinase 1
JUN: Jun proto-oncogene
VEGFA: Vascular endothelial growth factor A
CASP3: Caspase 3
CCND1: Cyclin D1
HIV: Human immunodeficiency virus
PPI: Protein–protein interaction
GO: Gene ontology
KEGG: Kyoto encyclopedia of genes and genomes
MEM: Minimum essential medium
SD: Sprague Dawley
YFJP-L: YFJP low-dose group
YFJP-H: YFJP high-dose group
EdU: 5-Ethynyl-2′-deoxyuridine
PBS: Phosphate buffer saline
qRT-PCR: RNA extraction and quantitative reverse transcription-polymerase chain reaction
cDNA: Complementary DNA
ANOVA: Analysis of variance
