# Supplementary material for: Molecular targets of Yangyin Fuzheng Jiedu Prescription in the treatment of hepatocellular carcinoma based on network pharmacology analysis
Source: Cancer Cell Int. 2020 Nov 9;20:540. doi: 10.1186/s12935-020-01596-y (PMC7650191; doi:10.1186/s12935-020-01596-y)
Supplement: Supplementary file 1 — Additional file 1. Additional tables. [file 12935_2020_1596_MOESM1_ESM.docx]

**Table S1.** The primer sequences.

| Gene name | Primer sequences (5’-3’) | |
| --- | --- | --- |
| TP53 | sense | CTGCTCAGATAGCGATGGTCTG |
|  | antisense | TTGTAGTGGATGGTGGTACAGTCA |
| CCND1 | sense | AGGAACAGAAGTGCGAGGAG |
|  | antisense | CACAGAGGGCAACGAAGGT |
| EGFR | sense | ATAGACGCAGATAGTCGCCCA |
|  | antisense | GCATTCTTTCATCCCCCTGA |
| EGF | sense | GACGCCTGTCTGMCCAGGA |
|  | antisense | CGA T AGCAGCTTCTGAGTCC |
| VEGFA | sense | CTACCTCCACCATGCCAAGT |
|  | antisense | GCAGTAGCTGCGCTGATAGA |
| JUN | sense | TCCCCCAGCTATCTATATGCAAT |
|  | antisense | TCACAGCACATGCCACTTGA |
| IL-6 | sense | GTCAACTCCATCTGCCCTTCAG |
|  | antisense | GGTCTGTTGTGGGTGGTATCCT |
| PTGS2 | sense | CAGCACTTCACGCATCAGTT |
|  | antisense | CGCAGTTTACGCTGTCTAGC |
| AKT1 | sense | GAAGGACGGGAGCAGGC |
|  | antisense | TGTACTCCCCTCGTTTGTGC |
| MAPK1 | sense | TGGATTCCCTGGTTCTCTCTAAAG |
|  | antisense | GGGTCTGTTTTCCGAGGATGA |
| ESR1 | sense | TCAGATAATCGACGCCAGGGTG |
|  | antisense | CACTTCGTAGCATTTGCGGAGCC |
| CASP3 | sense | GACTCTGGAATATCCCTGGACAACA |
|  | antisense | AGGTTTGCTGCATCGACATCTG |
| GAPDH | sense | GAAGGTGAAGGTCGGAGT |
|  | antisense | GAAGATGGTGATGGGATTTC |

Note: TP53, tumor protein p53; CCND1, cyclin D1; EGFR, epidermal growth factor receptor; EGF, epidermal growth factor; VEGFA, vascular endothelial growth factor A; JUN, jun proto-oncogene; IL-6, interleukin 6; PTGS2, prostaglandin-endoperoxide synthase 2; AKT1, AKT serine/threonine kinase 1; MAPK1, mitogen-activated protein kinase 1; ESR1, estrogen receptor 1; CASP3, caspase 3; GAPDH, glyceraldehyde-3-phosphate dehydrogenase.

**Table S2.** Cluster of hepatocellular carcinoma PPI network.

| Cluster | Score | Nodes | Edges | Genes |
| --- | --- | --- | --- | --- |
| 1 | 72.039 | 104 | 3710 | MAPK1, IL4, ALB, CXCL8, BCL2L1, CASP3, TP53, HSPA4, MMP2, SMAD2, HDAC1, CDKN1B, CDKN2A, TIMP1, MMP9, MMP14, ESR1, FOXP3, KDR, CASP8, BMP4, CAV1, IL1B, IL18, FOS, JUN, SNAI1, ICAM1, TLR9, SMAD3, FLT1, SMAD4, VEGFA, SPP1, CD44, PTEN, IL2, TNF, IL10, CDK2, ZEB1, CDKN1A, TGFB1, POU5F1, SOX2, FOXO3, HRAS, SERPINE1, PLAU, TLR2, CCNB1, FOXO1, IGF1R, IGF1, MMP7, PPARG, IL17A, CXCL12, CXCR4, PTGS2, STAT3, FASLG, MMP1, HIF1A, HSP90AA1, VCAM1, FGF2, TLR3, FN1, KRAS, CSF2, LEP, GAPDH, MTOR, CCL2, HNF4A, MDM2, IFNG, CXCL10, CCL5, MMP3, MPO, CDH1, MMP13, CRP, STAT1, SHH, EGF, EGFR, HGF, MAPK14, MET, SOCS3, SRC, AR, EGR1, HMOX1, ERBB2, NOTCH1, TLR4, CTNNB1, MAPK8, APOE, IL6 |
| 2 | 24.42 | 82 | 989 | TEK, ANGPT1, BMI1, EZH2, RELA, KRT19, CD24, SP1, STK11, TXN, EPO, CAT, XIAP, SOD2, IRF1, PDGFB, CDK6, SOCS1, ABCG2, MUC1, TIMP2, IGF2, GSK3B, AKT1, CDK4, CDKN3, TERT, ITGB1, RAF1, THY1, CREBBP, CDH17, FAS, BECN1, GJA1, TWIST1, AFP, IL1A, HDAC2, NANOG, ELANE, TNFSF10, CCNA2, FOXM1, RB1, CCND1, CCNE1, GLI1, SIRT1, CD274, PIK3CA, PTK2, MCL1, CDC25A, CDK1, CD34, PROM1, IRS1, EPCAM, ATM, CCR7, CHEK1, MKI67, NFKBIA, IGFBP3, NFKB1, MAPK3, AKT2, PTPN11, HMGB1, DNMT1, PXN, MYC, NRAS, PARP1, KLF4, ZEB2, SLC2A1, MYCN, WNT1, JAG1, IL6R |
| 3 | 6 | 6 | 15 | HLA-A, HLA-DQA1, HLA-B, HLA-DQB1, HLA-C, HLA-DRB1 |
| 4 | 5.455 | 12 | 30 | SMARCA2, G6PC, IGFBP1, APOB, LGALS1, HSP90B1, GOLM1, DNMT3B, NR0B2, FASN, CP, GPC3 |
| 5 | 5.448 | 30 | 79 | CDKN1C, MLH1, SFRP1, MSH2, SERPINA1, COL18A1, NQO1, PTTG1, TGFA, INSR, SQSTM1, SPARC, MGMT, RRM2, SFN, ITGA5, ARID1A, CLU, PCNA, IDH2, IDH1, BRAF, GNAS, RASSF1, MAD2L1, DNMT3A, RUNX3, CKS1B, TYMS, BAX |
| 6 | 5.348 | 47 | 123 | PDGFRA, IFNA1, AURKB, IL2RA, F2, DDIT3, SMAD7, YAP1, PTCH1, TGFBR2, DKK1, AREG, ABCB1, VDR, BIRC5, CEBPA, CEBPB, GPT, KLRK1, AXIN1, FGFR1, ANXA2, NFE2L2, IFNB1, VTN, TNFRSF10B, CFLAR, GPR29, PKM, STAT4, HSPA5, TIMP3, SKP2, VIM, CDKN2B, PLK1, E2F3, ANGPT2, AXIN2, FGF3, FGF4, FBXW7, AHR, E2F1, AURKA, HSPB1, NOTCH3 |
| 7 | 4.333 | 7 | 13 | GSTM1, NR1I2, CYP1A2, CYP3A4, ABCC2, ABCC1, DPYD |
| 8 | 3.333 | 4 | 5 | HP, TFRC, LCN2, LDLR |
| 9 | 3.333 | 4 | 5 | XRCC3, OGG1, CYP1A1, EPHX1 |
| 10 | 3 | 3 | 3 | APEX1, NME1, PRDX1 |
| 11 | 3 | 3 | 3 | ARID2, ARID1B, SETD2 |

**Table S3.** Cluster of herbal formula-disease PPI network.

| Cluster | Score | Nodes | Edges | Genes |
| --- | --- | --- | --- | --- |
| 1 | 29.879 | 34 | 613 | BCL2L1, CCND1, VEGFA, AKT1, EGFR, RELA, CCNB1, MMP3, HSPB1, PLAU, IL1B, CASP3, ICAM1, Quercetin, JUN, ERBB2, STAT1, HIF1A, MMP1, PTGS2, AR, TP53, IL6, TNF, EGF, IL10, MAPK1, IGF1, MMP9, ESR1, MMP2, MDM2, CDKN1A, FOS |
| 2 | 2.667 | 4 | 4 | IRF1, RB1, TERT, AHR |
