# Supplementary figures and images for: Molecular targets of Yangyin Fuzheng Jiedu Prescription in the treatment of hepatocellular carcinoma based on network pharmacology analysis
Source: Cancer Cell Int. 2020 Nov 9;20:540. doi: 10.1186/s12935-020-01596-y (PMC7650191; doi:10.1186/s12935-020-01596-y)

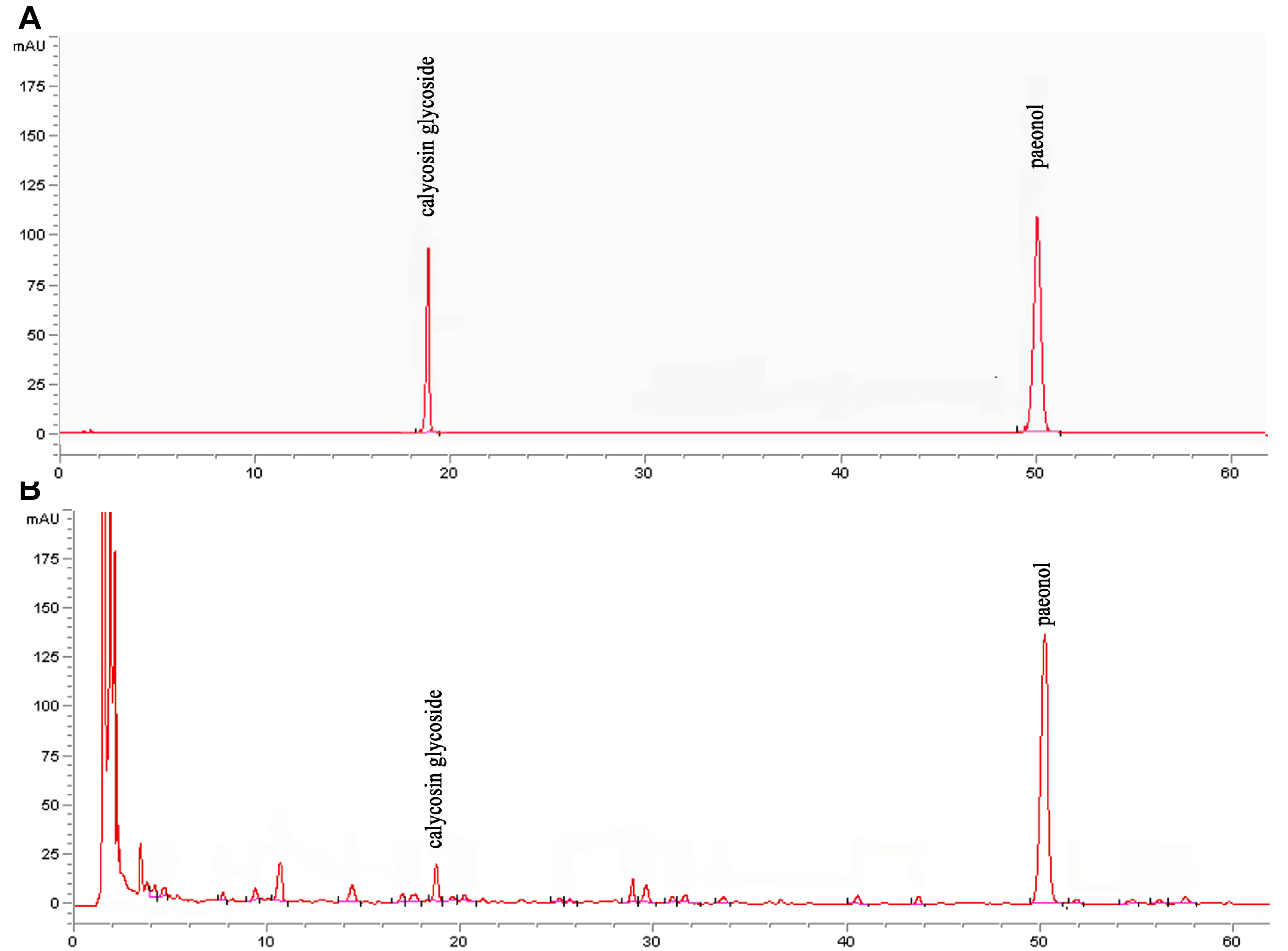

Supplement: Supplementary file 2 — Additional file 2: Figure S1. HPLC analysis of YFJP. A: Standard control mixture; B: UV tracer of HPLC analysis of YFJP. [file 12935_2020_1596_MOESM2_ESM.jpg]
